# Insanity from Hunger, Fear and Suffering

**Published:** 1846-02

**Authors:** 


*Insanity from hunger, fear and suffering.*—In Captain Fremont's interesting narrative of the second exploring expedition to Oregon, we find the following: "On the first of March, one of the men named Derosier who had volunteered to bring up Capt. Fremont's horse, had not come back to the camp and uneasiness was felt at his absence. He however made his appearance in the evening. He came in, and sitting down by the fire, began to tell us where he had been. He imagined he had been gone several days, and thought we were still at the camp, where he had left us, and we were pained to see that his mind was deranged. It appeared that he had been lost in the mountain, and hunger and fatigue, joined to weakness of body and fear of perishing in the mountains, had crazed him. The times were severe, when stout men lost their minds from extremity of suffering. The fate of this poor fellow was a melancholy one. On the 23d of March, he wandered away, and has not since been heard of."—*American Journal of Insanity.*

